# Clinical Study for Safety Evaluation of GXN Tablets Combined with Aspirin in Long-Term Treatment of Coronary Heart Disease

**DOI:** 10.1155/2021/6658704

**Published:** 2021-06-07

**Authors:** Mingjie Zi, Ruiqi Li, Fang Lu, Qingna Li, Yang Zhao, Huijuan Jia, Mingyue Sun, Qiaoning Yang, Panbo Qiu, Eryang Wei, Shuge Wang, Rui Li, Wantong Zhang, Weiyi Cao, Yanling Li, Hao Xu, Rui Gao

**Affiliations:** ^1^Institute of Clinical Pharmacology, Xiyuan Hospital of China Academy of Chinese Medical Sciences, Beijing 100091, China; ^2^National Clinical Research Center for Chinese Medicine Cardiology, Beijing 100091, China; ^3^NMPA Key Laboratory for Clinical Research and Evaluation of Traditional Chinese Medicine, Beijing 100091, China; ^4^Graduate School, Beijing University of Chinese Medicine, Beijing 100029, China; ^5^Cardiovascular Diseases Center, Xiyuan Hospital of China Academy of Chinese Medical Sciences, Beijing 100091, China

## Abstract

**Background:**

GXN tablets are composed of Danshen and Chuanxiong, with the functions of activating blood circulation, removing blood stasis, invigorating the pulse, and nourishing the heart, which are used for CHD patients with stable exertional angina Grade I or II (according to traditional Chinese medicine, it is a syndrome of heart and blood stasis with chest pain and dark purple lips and tongue). Clinical trials have shown satisfactory effects on coronary heart disease (CHD). 90.6% of Chinese patients with CHD use both Western medicine and Chinese medicine with the latter being thought to promote blood circulation and remove blood stasis. Some researchers doubt that the combination of Chinese medicine increases the risk of bleeding. The main objective of this study is to observe the safety of long-term use of Guanxinning (hereafter referred to as GXN) tablets combined with aspirin.

**Methods:**

The study population is patients with CHD after percutaneous coronary intervention (PCI). Randomization was performed for patients with stable CHD who received dual antiplatelet therapy (DAPT) with aspirin plus clopidogrel or ticagrelor for more than 12 months and then switched to the treatment with aspirin alone for 1 month. This study includes a total of 3,595 subjects in 63 hospitals. The experimental group took aspirin orally (100 mg, 1 time/day) + GXN tablets (0–6 months: 4 tablets/time, 3 times/day; 7–12 months, 4 tablets/time, 2 times/day), and the control group received oral aspirin (100 mg, 1 time/day). Major observation indicators are the incidence of bleeding events, adverse events (AEs), and adverse reactions. The primary endpoint indicators are the incidence of major adverse cardiovascular and cerebrovascular events (MACCE) and the MACCE composite endpoint.

**Results:**

A total of 31 cases of symptomatic bleeding were found in the two groups, including 21 cases (0.98%) in the experimental group and 10 cases (0.86%) in the control group; the difference between the two groups was not statistically significant. There were 29 cases (1.35%) of bleeding not reaching BARC type 1 in the experimental group. No attention was paid to the laboratory indicators in the control group during the trial process, so the bleeding as a laboratory indicator between the two groups was not comparable. For BARC type 3–5 bleeding events, there were 3 cases in the experimental group (0.139%) and 2 cases in the control group (0.172%); the difference between the two groups was not statistically significant and not clinically significant. During the trial period, there were a total of 255 cases of adverse reactions in 208 subjects with an incidence of 6.57% (141/2146) in the experimental group and 5.77% (67/1161) in the control group, and the *P* value was 0.5021; the difference between the two groups was not statistically significant. According to the analysis, the adverse reactions with a statistically significant difference between the two groups were gastrointestinal diseases, with the incidence in the experimental group being higher than that in the control group, and the main manifestations were gastrointestinal symptoms. There was no statistical difference in other types of adverse reactions between the two groups. In the trial period, there were 10 cases of serious adverse reactions, including 5 cases in the experimental group (5/2146, 0.23%) and 5 cases in the control group (5/ 1161, 0.43%), the *P* value was 0.3351; the difference in the incidence between the two groups was not statistically significant.

**Conclusion:**

For CHD patients with heart-blood stasis syndrome, the combination of aspirin and GXN tablets in the experimental group did not increase the incidence of bleeding events, nor did it increase the risk of bleeding of types 3–5 defined by BARC. Except for the increase in gastrointestinal symptoms, there was no increase in other adverse reactions in the experimental group. This trial is registered with Registration no. ChiCTR-IIR-17010688.

## 1. Introduction

The ESC Guidelines on Cardiovascular Prevention point out that long-term use of aspirin is the basic measure for secondary prevention of CHD. However, it is reported that 90.6% patients with CHD, in addition to aspirin as a preventive drug, take Chinese medicine [1]. The clinical drug data in the real world also show that the most commonly used preventive drug for patients with CHD is Chinese medicine for promoting blood circulation and removing blood stasis [2], although some research literature reported that the combination of Chinese medicine can increase the risk of bleeding [3]. GXN tablets with the main ingredients of Danshen and Chuanxiong and the effects of activating blood circulation, removing blood stasis, invigorating the pulse, and nourishing the heart are used for CHD patients with stable exertional angina Grade I or II (which is considered by traditional Chinese medicine a syndrome of heart and blood stasis with chest pain and dark purple lips and tongue). The package insert has the following information: “the patient may have stomach pain after taking the medicine.” A proposal is that the clinical dosage of GXN tablets should be 4 tablets at a time, 3 times a day [4]. In this study, the Chinese patent medicine GXN tablets, which have effects of promoting blood circulation and removing blood stasis, when used with aspirin, are compared with oral aspirin tablets alone in the incidence of bleeding events and the incidence of adverse events (AEs). The aim of this study is to evaluate the safety of GXN tablets combined with aspirin and explore the ideas and methods for safety evaluation of the combined application of Chinese and Western medicine.

## 2. Methods

### 2.1. Design

This is a stratified, cluster-randomized, and multicenter study adopting a large sample size. A total of 63 hospitals are included, and cluster randomization is performed according to the attributes of hospitals of integrated Chinese and Western medicine. This trial has been registered in the Chinese Clinical Trial Registry (Registration no.: ChiCTR-IIR-17010688), and the trial protocol has been published in the Trials journal [5]. The protocol is formulated by Xiyuan Hospital of China Academy of Chinese Medical Sciences. The institute of clinical pharmacology of our hospital is responsible for data management and setups of a data safety supervision committee and an endpoint event determination committee. Data analysis is performed by an independent statistical analysis unit that was unaware of the trial-group assignments. This study is conducted by a contract research organization (CRO) and is approved by the ethics committee of Xiyuan Hospital, with all patients signing an informed consent form before treatment (Approved No.: 2016XL0102). The study was conducted in accordance with the principles of Good Clinical Practice and the Declaration of Helsinki [6].

### 2.2. Patients

Eligible patients must meet inclusion criteria: (1) patients with stable CHD after PCI subject to DAPT (aspirin + clopidogrel or ticagrelor) for more than 12 months and then switched to the treatment with aspirin alone for 1 month; (2) patients with TCM syndrome of blood stasis (diagnosed if there is one of the following three conditions: dark purple or ecchymoses, petechiae; cyanotic or dark lips; sublingual varicose veins); (3) age of 18 to 80, regardless of gender; and (4) volunteers who sign an informed consent form. Exclusion criteria included the following: (1) patients with heart function IV (NYHA); (2) patients combined with uncontrolled grade III hypertension (systolic blood pressure ≥180 mmHg and/or diastolic pressure ≥110 mmHg), patients with severe cardiopulmonary insufficiency, patients with severe cardiac arrhythmia, patients with severe primary diseases in the liver, kidney, and hematopoietic system or patients with serious diseases (such as tumor) and mental illnesses that affect their survival; (3) patients who have the other clinical conditions that may increase the risk of bleeding; (4) patients with abnormal liver and kidney function indicators (ALT, AST, and Scr exceeding the upper limit of the normal reference range by 1.5 times); (5) patients with allergic constitution or allergies to foods and drugs, or patients known to be allergic to the test drug (including its ingredients); (6) patients with pregnancy potential or pregnant or lactating women; and (7) patients in other clinical trials within the past 1 month.

### 2.3. Interventions

This study adopted the method of hospital stratification and cluster randomization, in which a hospital only included the experimental group or the control group. There were a total of 63 hospitals (Chinese medicine hospitals: Western medicine hospitals = 2 : 1). The participating hospitals (clusters) with 3600 patients are firstly stratified by type of medication delivery (Western medical or traditional Chinese medicine hospitals), and then hospitals in each stratification are randomly allocated in a 2 : 1 ratio by a computer-generated, centrally controlled randomization schedule to treatment (24 hospitals with 2400 patients) or control (12 hospitals with 1200 patients) arms. All patients from one cluster will receive the same intervention. Though the patients and caregivers are not blinded, the outcome assessors and data analysts are to be blinded to treatment allocation to minimize potential bias. The cases are assigned according to the proportion of the experimental group: control group = 2 : 1. Both groups continued to receive aspirin (100 mg once daily) and the other conventional treatment. On the basis of using the basic drugs, the experimental group additionally took GXN tablets four tablets each time, three times daily for the first 6 months, which was reduced to four tablets each time, twice daily for another 6 months. At 12 months, patients in the experimental group will stop GXNT, and both groups will continue aspirin monotherapy and the other conventional treatment for another 12 months. In accordance with the relevant diagnosis and treatment guidelines, it was allowed to take sublingual nitroglycerin during the acute attack of angina. During the trial period, no other similar drugs against antiplatelet aggregation and Chinese medicines for promoting blood circulation and removing blood stasis were taken, and all drugs taken were recorded truthfully.

Safety was observed in the experimental group with combined medication for 1 year: visits were made before enrollment and 1 month, 3 months, 6 months, and 12 months after medication. Safety was observed in the control group with aspirin alone for 1 year: visits were made before enrollment and 1 month, 3 months, 6 months, and 12 months after medication. The experimental group was tested for laboratory indicators before enrollment and 1 month, 3 months, 6 months, and 12 months after medication; the control group was tested only before enrollment (the coagulation function was monitored once 3 months after taking the drug). Bleeding events, AEs, adverse reactions, and endpoint indicators were observed and recorded at each observation point.

### 2.4. Observation Indicator

The main observation indicator is safety, including the incidence of bleeding events, adverse events, and adverse reactions. In the evaluation of bleeding events, the unified definition of bleeding proposed by the BARC was used [7]. See the attached table for the definition of bleeding. The primary endpoint is the MACCE, namely, the incidence of MACCE composite endpoint. Cardiovascular death, stent thrombosis, nonfatal myocardial infarction, revascularization, ischemic stroke, and hospitalization due to unstable angina pectoris are observation indicators of efficacy in this trial. These events are not recorded as adverse events. Accordingly, hospitalization and deaths caused by these events are not recorded and reported as SAEs. The endpoint event committee of this trial convened a meeting in time to determine the endpoint event.

### 2.5. Statistical Analysis

The primary endpoint of this study is the MACCE compound incidence under aspirin anticoagulation treatment. According to the literature, it is assumed that the incidence of MACCE is 12.0% after the subjects included in this trial continued to undergo aspirin intervention for 12 months, that the incidence of MACCE is reduced by 21% after 12 months of adding GXN tablets, that the power of the test is 80%, that the false-positive error rate is controlled at 2.5% (unilateral), and that the cases are assigned to two groups in a 2 : 1 ratio. According to the above assumption, after a 12-month recruitment period and a 12-month follow-up period, it was planned to enroll 3,600 subjects (2,400 in the experimental group and 1,200 in the control group), with expected 540 cases of endpoint events (332 cases in the experimental group and 209 cases in the control group). Baseline data are analyzed on a full analysis set (FAS), and laboratory test data and adverse event and adverse reaction data are analyzed by safety set (SS). The authenticity and integrity of the data are ensured by the independent data security inspection committee.

The general hypothesis test is a two-sided test, with *P*=0.05, and an appropriate statistical method is selected for statistical description according to the normality of the data. Outliers are determined according to the judgment of clinical professionals and statistical criteria. When major efficacy data is missing, the method of data supplementation is determined from a statistical and professional perspective. The mean, standard deviation, median, minimum, and maximum are calculated in the description of quantitative indicators. The classification indicators are described by the case number and percentage of each type. Differences of measurement data are compared with group *t*-test, paired *t*-test, Kruskal–Wallis H test, and Wilcoxon signed-rank test. Count data are analyzed using the chi-square test and Fisher's exact test. Kruskal–Wallis H test is used for ranked data. CMH chi-square test is used when considering the central effect.

The Cox proportional hazard model is used to adjust for other risk factors affecting the incidence of cardiovascular events, compare the hazard/risk of the primary endpoint event between groups, and report the risk ratio of bleeding events in each group (95% CI). MACCE occurrence time: the Kaplan–Meier method is used to calculate the occurrence time for 25%, 50% (median), and 75% of cardiovascular events in the two groups during the trial period, respectively, and survival curves are drawn for the two groups. The cases of adverse events during the trial are described in detail; the incidences of adverse events between the two groups are summarized and compared; the two groups are compared by Fisher's exact test or chi-square test. Laboratory data: statistical description is mainly used to analyze the changes before and after treatment. The average values of each laboratory data before and after treatment are compared. The normal and abnormal changes of laboratory indicators before and after treatment are described in detail. SAS9.4 software is used for statistical analysis.

## 3. Results

### 3.1. Subjects

From March 1, 2017, to December 28, 2018, a total of 3,595 subjects were enrolled in 63 hospitals. Nine patients did not meet the requirements for informed consent, so there were 3,586 patients in FAS. 279 patients did not take medicine or did not have the laboratory examination and adverse event records, so there were 3,307 patients in SS. The analysis of baseline data was performed on FAS, and the laboratory test data and adverse events and adverse reaction data were analyzed by SS.

The baseline characteristics of subjects in the two groups are comparable (Table 1). The median duration of medication in the experimental group is 52.30 weeks (interquartile range (IQR), 51.70∼53.10).

### 3.2. Bleeding

There were 66 bleeding events in 60 cases of subjects in the two groups, including 53 cases in the experimental group (29 cases of bleeding defined by laboratory indicators, 1.35%) and 13 cases in the control group; the difference was statistically different.

According to the protocol, the subjects in the experimental group underwent three major routine tests, liver function test, coagulation function test, and electrocardiogram test before enrollment and 1 month, 3 months, 6 months, and 12 months after enrollment; the subjects in the control group only underwent the above tests before enrollment and the coagulation function test in the third month. Therefore, the overall comparison between the two groups was not suitable in terms of laboratory tests, and the difference was not comparable. Bleeding was divided into inspection-indicated bleeding and noninspection-indicated bleeding. That is, analysis was performed after the results of various tests were excluded. At each follow-up point, there was no difference between the two groups. There were 29 cases (1.35%) of laboratory indicator defined bleeding (not reaching BARC type 1) in the experimental group. There was no attention paid to laboratory indicators in the trial process in the control group, so the two groups were not comparable in terms of laboratory indicator defined bleeding.

Noninspection-indicated bleeding: there were 4 cases (0.2%) in the experimental group and 0 cases in the control group within 1 month of medication; 11 cases in the experimental group (0.5%) and 4 cases in the control group (0.3%) within 3 months of medication; 14 cases (0.7%) in the experimental group and 6 cases in the control group (0.5%) within 6 months of medication; 20 cases in the experimental group (0.9%) and 10 cases in the control group (0.9%) within 12 months of medication. It can be seen that there is no statistical difference between the two groups in the incidence of bleeding events at these follow-up points.

It is shown from the symptoms of bleeding events in subjects in the two groups that the highest number of bleeding events occurred in various inspections: 29 cases (1.35%) in the experimental group that did not reach the BARC type 1 bleeding. Noninspection-indicated bleeding: a total of 31 cases of symptomatic bleeding occurred in the two groups, including 21 cases (0.98%) in the experimental group and 10 cases (0.86%) in the control group. There was no statistically significant difference between the two groups. After inspection-indicated bleeding was excluded, the difference between the two groups was not statistically significant in terms of bleeding caused by diseases in the gastrointestinal tract, respiratory system, respiratory system, chest, mediastinum, eye, neurological system, kidney and urinary system, blood, and lymphatic system. The difference between the two groups also was not statistically significant in the incidence of bleeding events. BARC type 3–5 bleeding events: 3 cases (0.139%) in the experimental group and 2 cases (0.172%) in the control group (cerebral hemorrhage and ocular fundus hemorrhage); the difference between the two groups was not statistically and clinically significant.

### 3.3. Adverse Events

During the trial period, there were 2,285 adverse events (AEs) in 1,206 subjects. The incidence of AEs in the experimental group was 39.24% (842/2146), with 84 subjects withdrawing from the trial due to AEs. The incidence of AEs in the control group was 31.35% (364/1161); the difference was statistically significant.

From the analysis of the symptoms of adverse events by frequency (only the symptoms or diseases with a frequency of more than 3%), it can be seen that the main diseases were as follows. For infections, there were 249 cases (11.6%) in the experimental group and 168 cases (14.47%) in the control group; the control group was higher than the experimental group and the difference was statistically significant. For gastrointestinal diseases, there were 187 cases (8.71%) in the experimental group and 68 cases (5.86%) in the control group; the experimental group was higher than the control group and the difference was statistically significant. For various inspections, there were 176 cases in the experimental group (8.2%) and 6 cases in the control group (0.52). For heart diseases, there were 115 cases in the experimental group (5.36%) and 36 cases in the control group (3.1%); the experimental group was higher than the control group and the difference was statistically significant. For various neurological diseases, there were 89 cases (4.15%) in the experimental group and 40 cases in the control group (3.45%); the difference was not statistically significant.

For inspection-indicated AEs, there were 176 cases (8.2%) in the experimental group and 6 cases (0.52%) in the control group. For noninspection-indicated AEs, there were 666 cases (31.04%) in the experimental group and 358 cases (30.83%) in the control group.

During the trial, a total of 367 subjects had 458 serious adverse events (SAEs). The incidence of SAEs was 12.12% (260/2146) in the experimental group and 9.22% (107/1161) in the control group.

### 3.4. Adverse Reactions

During the trial, 208 subjects had 255 adverse reactions. The incidence of adverse reactions in the experimental group was 6.57% (141/2146), and the control group 5.77% (67/1161). There was no statistically significant difference in the overall incidence of adverse reactions between the two groups.

With inspection-indicated adverse reactions excluded, the difference between the two groups was statistically significant only in the first month. Specifically, there were 59 cases (2.7%) in the experimental group and 15 cases in the control group (1.3%) within 1 month of medication; 86 cases (4.0%) in the experimental group and 38 cases (3.3%) in the control group within 3 months of medication; 106 cases (4.9%) in the experimental group and 49 cases (4.5%) in the control group within 6 months of medication; 120 cases (5.6%) in the experimental group and 64 cases (5.5%) in the control group within 12 months of medication.

The frequency of occurrence of adverse reactions is as follows (in descending order): gastrointestinal diseases: 57 cases (2.66%) in the experimental group and 7 cases (0.6%) in the control group; various inspections: 23 cases (1.07%) in the experimental group and 1 case in the control group (0.09); hepatobiliary system diseases: 19 cases in the experimental group (0.89%) and 1 case in the control group (0.09); systemic diseases and various reactions at the administration site: 14 cases in the experimental group (0.65%) and 4 cases (0.34%) control group; and infections: 11 cases (0.51%) in the experimental group and 33 cases (2.84%) in the control group.

In gastrointestinal diseases, the difference between the two groups was statistically significant; the frequency of occurrence in the experimental group was higher than that in the control group, and the main manifestation was gastrointestinal symptoms. There was no statistical difference in other adverse reactions between the two groups.

Inspection-related adverse reactions: there were 23 cases (1.07%) in the experimental group and 1 case (0.09%) in the control group. Noninspection-related adverse reactions: there were 118 cases (5.5%) in the experimental group and 68 cases (5.93%) in the control group.

During the trial, a total of 10 subjects had 10 serious adverse reactions. There was no statistically significant difference in the incidence of serious adverse reactions between the two groups.

### 3.5. Laboratory Indicators

Abnormal changes in laboratory indicators before and after medication: the subjects in the experimental group were subject to the three major routine tests, liver function test, coagulation function test, and electrocardiogram test before enrollment and 1 month, 3 months, 6 months, and 12 months after enrollment; the subjects in the control group only underwent the above tests before enrollment and the coagulation function test in the third month. That is, unlike the experimental group, the control group did not undergo the three major routine tests, blood biochemistry test, and electrocardiogram test in other time points. Therefore, only the coagulation function was compared between the two groups; in the blood biochemical parameter, the difference was clinically significant before and after medication. Moreover, the differences mostly happen among liver and kidney function abnormalities, which can be caused by lots of combined medications; thus, their relations with GXNT cannot be verified.

There were reports of abnormalities in liver function (ALT, AST, and GGT) and renal function (BUN and Scr) of subjects in the experimental group. At the end of the 12 months, the number of abnormalities with clinical significance of ALT, AST, GGT, BUN and Scr was, respectively, 4 (0.17%), 2 (0.08%), 1 (0.04%), 4 (0.17%), and 8 (0.33%). As there were no data from the control group, comparisons between the two groups could not be made.

In the coagulation function before enrollment and three months after enrollment, the difference between the two groups was not statistically significant.

### 3.6. Combined Medication

Drugs for combined use are mainly as follows: digestive tract drugs (e.g., oral drugs, drugs for diseases related to gastric acid secretion, and drugs for constipation and diabetes), drugs for blood and hematopoietic organ diseases (e.g., antibleeding drugs, antianemia drugs, blood substitutes and perfusion fluids), drugs for cardiovascular system diseases (e.g., antithrombotic drugs, antihypertensive drugs, diuretics, peripheral vasodilators, vascular endothelial protection agent, *β*-receptor blockers, and blood lipid regulators), dermatological drugs (e.g., antifungal drugs, skincare agents, wound and ulcer drugs, and antipruritic drugs), genitourinary system drugs (e.g., gynecological anti-infectives and sterilization drugs, sex hormones, and reproductive system regulators), hormone preparations excluding sex hormones and insulin (e.g., pituitary and hypothalamic hormones and similar drugs, systemic corticosteroids, and thyroid drugs), antitumor drugs and immunomodulators (e.g., antitumor drugs, endocrine therapy, immune promoting drugs, and immunosuppressive drugs), musculoskeletal system drugs, nervous system drugs, respiratory system drugs, and TCM drugs. The statistical results showed that the difference between the two groups regarding the types and numbers of the combined medication during the trial was not statistically significant.

## 4. Discussion

Regarding the incidence of bleeding events, this study is different from previous reports in the literature. This study shows that under the guidance of accurate syndrome differentiation in CHD patients with blood stasis syndrome, the use of GXN tablets combined with aspirin for a long time did not increase the risk of type 3–5 bleeding events and did not increase the incidence of bleeding events, despite bleeding events observed (e.g., abnormal occult blood indicator, type 1-2 bleeding), compared with the control group. Bleeding events occurred most frequently in various inspections in the experimental group, such as stool occult blood test, urine routine test, and urine occult blood test, all of which were all mild type 1-2 bleeding events. The control group did not perform related tests in accordance with the protocol, without records of such types of bleeding, so the two groups are not comparable.

In gastrointestinal diseases, the difference between the two groups was statistically significant; the frequency of occurrence in the experimental group was higher than that in the control group. There was no statistical difference in other adverse reactions between the two groups. Therefore, the use of GXN tablets combined with aspirin might increase gastrointestinal symptoms but did not significantly increase the risk of other adverse reactions.

Regarding laboratory indicators, there are no data in the control group, so comparisons between the two groups cannot be made. At the same time, this is a real-world study, the subjects in the two groups have underlying diseases, and each subject is treated comprehensively. Abnormal liver and kidney function cannot be viewed only from before and after treatment, since many drugs in combined use may affect liver and kidney function. It is currently impossible to infer the relationship with the experimental drug from the current data; instead, it is necessary to further observe the situation after stopping the medication. There was no significant difference in coagulation between the two groups before enrollment and 3 months after enrollment, indicating that long-term oral administration of GXN tablets based on aspirin did not cause abnormal blood coagulation function.

Among basic drugs in combined use in the two groups, there are drugs such as statins that have an impact on liver function [8]. In the trial, there was a rise in laboratory indicators observed, such as liver function and renal function, but we cannot say this is directly related to the experimental drug. More data are needed.

Our study team searched a large number of studies on the efficacy of GXN in the treatment of CHD and angina pectoris. We found that most of the experimental groups in these clinical studies took conventional treatment plus GXN, while most of their control groups had injections, such as Xiangdan injection and Danshen injection or simply receiving conventional treatments. Moreover, these studies have the following characteristics: small sample size (about 50 to 200 cases), a short observation period (mostly 1-2 weeks), a lack of objective evaluation criteria on curative effect, and no attention to safety indicators [9–18].

The safety observation indicators of this study are the incidence of bleeding events and adverse reactions. The following aspects need to be considered.

### 4.1. Patients

Patients should be representative of the target population and suitable for observation of safety. For Chinese medicine, the accuracy of syndrome differentiation should be considered. Combined with the main results of this study, it can be said that some increases in bleeding events may be related to the inaccurate selection of the medication population. This kind of Chinese medicine should be applied to patients with blood stasis syndrome. Among the population with adverse reactions, attention should be paid to the accuracy of syndrome differentiation or further clinical research should be carried out to analyze the incidence of adverse reactions under different syndromes. It should be emphasized that the clinical application of Chinese medicine should be applied under the guidance of TCM theory.

There is a difference in the treatment regimen of Chinese medicine combined with aspirin between Chinese and the Western medicine hospitals, and the sample size of this study is large. Thus, cluster randomization can ensure the balance of Chinese and Western medicine hospitals. The random assignment of cases at a ratio of 2 : 1 between the experimental group and the control group can effectively ensure intergroup balance and within-group consistency. Cluster randomization in postmarketing study is also one of the innovations of this study.

### 4.2. Treatment and Follow-Up

The treatment course studied on existing postmarketed oral Chinese patent medicines for CHD is generally 6 months to 1 year. Aspirin as a secondary preventive drug is used for a long period of time. This study observes the safety of the combined use of GXN and aspirin. Considering the safety during the medication, hoping to answer the safety questions while answering whether it is effective, the overall observation period of this study is two years. In order to intensively observe the experimental group, we record AEs (including bleeding) and adverse reactions and detect laboratory indicators at 1 month, 3 months, 6 months, and 12 months after taking the drug to ensure that the subjects' condition and hematological changes are under the effective supervision and that the safety of subjects is fully ensured. Due to the control group only orally taking aspirin, the study minimizes the intervention by only performing baseline tests before enrollment and the coagulation function test in the third month after enrollment to ensure the safety of subjects. However, there is a shortcoming of the study found in this analysis. The inspection of laboratory indicators in the control group should be added to the time point of follow-up to clarify the factors when laboratory adverse events occur.

## 5. Conclusion

For CHD patients with heart-blood stasis syndrome, the combination of aspirin and GXN tablets in the experimental group did not increase the incidence of bleeding events, nor did it increase the risk of bleeding of type 3–5 defined by BARC. Except for the increase in gastrointestinal symptoms, there was no increase in other adverse reactions in the experimental group.

## Figures and Tables

**Table 1:** Baseline characteristics.

Indicators	Experimental group (*N* = 2399)	Control group (*N* = 1187)
Age (years)	62.3 ± 8.9	62.2 ± 9.0
Female	542 (22.6)	280 (23.6)

*Nationality*		
Han	2357 (98.5)	1142 (96.2)
Others	37 (1.5)	45 (3.8)

Height (cm)	166.56 ± 7.59	166.46 ± 7.78
Weight (kg)	70.48 ± 11.29	69.75 ± 11.41
BMI (kg/m^2^)	25.35 ± 3.31	25.10 ± 3.28
Respiration (times/min)	17.7 ± 1.8	18.3 ± 1.6
Resting heart rate (beats/min)	68.8 ± 9.3	70.5 ± 9.6
Body temperature (°C)	36.38 ± 0.32	36.52 ± 0.29
Systolic blood pressure (mmHg)	129.2 ± 14.2	130.9 ± 13.8
Diastolic blood pressure (mmHg)	77.5 ± 9.2	77.7 ± 9.4

*Medical history*		
History of current disease: PCI time	36.27 ± 26.22	32.13 ± 22.36
History of current disease: 12 months after PCI	2377 (99.7)	1181 (99.9)
History of allergy	234 (9.8)	103 (8.7)
History of other diseases	1873 (78.1)	971 (81.8)

*Antiplatelet therapy after PCI*		
Aspirin therapy	2067 (87.2)	1045 (89.8)
Clopidogrel therapy	1999 (84.3)	935 (80.3)
Ticagrelor therapy	84 (3.5)	137 (11.8)

Syndrome of heart-blood stasis	2379 (100.0)	1161 (99.6)

*Heart function (NYHA grade) at enrollment*		
I	1530 (64.2)	860 (73.6)
II	807 (33.9)	306 (26.2)
III	46 (1.9)	3 (0.3)
IV	0 (0.0)	0 (0.0)

## Data Availability

The data used to support the findings of this study are available from the corresponding author upon request.
